# Trophic Transfer of Cd, Cu, Pb, Zn, P and Se in Dutch Storage Water Reservoirs

**DOI:** 10.1007/s00244-023-01041-x

**Published:** 2024-01-20

**Authors:** Yvon J. M. Verstijnen, Esther C. H. E. T. Lucassen, Arco J. Wagenvoort, Henk A. M. Ketelaars, Gerard van der Velde, Alfons J. P. Smolders

**Affiliations:** 1 B-WARE Research Centre, Radboud University, Nijmegen, The Netherlands; 2 Department of Aquatic Ecology and Environmental Biology, Radboud Institute for Biological and Environmental Sciences (RIBES), Radboud University, Nijmegen, The Netherlands; 3 AqWa, Goes, The Netherlands; 4 Evides Water Company, Rotterdam, The Netherlands; 5 Rubiconsult, Dordrecht, The Netherlands; 6 Department of Animal Ecology and Physiology, Radboud Institute for Biological and Environmental Sciences, Radboud University (RIBES), Nijmegen, The Netherlands; 7 Naturalis Biodiversity Center, Leiden, The Netherlands; 8 Netherlands Centre of Expertise On Exotic Species (NEC-E), Nijmegen, The Netherlands

## Abstract

Heavy metals are naturally omnipresent in aquatic systems. Excess amounts of heavy metals can accumulate in organisms of pollution impacted systems and transfer across a food web. Analysing the food web structure and metal contents of the organisms can help unravel the pathways of biomagnification or biodilution and gain insight in trophic linkages. We measured heavy metals and other elements in mussel bank detritus and organisms of the Biesbosch reservoirs (the Netherlands) and linked those to stable isotopic signatures. The heavy metal contents (cadmium, copper, lead, and zinc) were often lowest in benthivorous, omnivorous and piscivorous species (mainly fish); whereas, phosphorus contents were lower in the autotrophs. Mussel bank detritus contained the highest amounts of heavy metals. The heavy metals were negatively correlated with δ^15^N values. For selenium no clear trend was observed. Furthermore, there was a negative correlation between fish length and some heavy metals. Based on all 20 analysed elemental contents, similarities between species became apparent, related to niche or habitat. This study confirms that elemental contents of species can differ between feeding guilds and/or species, which can be attributed to metabolic and physiological processes. The organisms in higher trophic levels have adaptations preventing metal accumulation, resulting in lower contents. Within the fish species biodilution occurs, as most metal contents were lowest in bigger fish. Overall, the metals did not seem to biomagnify, but biodilute in the food web. Metal analyses combined with isotopic signatures could thus provide insights in metal transfer and possible trophic linkages within a system.

**Supplementary Information:**

The online version contains supplementary material available at 10.1007/s00244-023-01041-x.

Heavy metals are naturally omnipresent in aquatic systems and several play a key role in the metabolism of organisms (Luoma 1983; Rainbow 2002). These are essential elements. Other metals are considered non-essential. Due to anthropogenic activities, such as mining and discharge of industrial and urban waste, excess amounts of heavy metals can enter aquatic systems, polluting streams, rivers, lakes and reservoirs (Mollema et al. 2015; Subotić et al. 2015; Schmutz and Sendzimir 2018). As metals can accumulate in the sediment, impacted systems often are sinks for heavy metals (Mollema et al. 2015; Schmutz and Sendzimir 2018).

Biota living in these systems can take up and accumulate metals from the sediment or water (e.g. Luoma 1983; Naimo 1995; Marentette et al. 2010; McEneff et al. 2017) and/or via their diet (e.g. Croteau et al. 2005; DeForest et al. 2007; Balzani et al. 2021). This is known as bioaccumulation, which is a well-known phenomenon (DeForest et al. 2007). When present in excess amounts, the uptake of non-essential or essential metals can influence the composition of biota, which can experience detrimental effects (e.g. Luoma 1983; Guilizzoni 1991; Fleeger et al. 2003; Smolders et al. 2003). Bioaccumulation can lead to further accumulation of the metals in the food web (known as biomagnification) and possibly adverse effects on biota (e.g. Vijver et al. 2004; Croteau et al. 2005; DeForest et al. 2007).

The eventual metal content of an organism is dependent on several biotic and abiotic factors, and is metal and species-specific (e.g. Luoma 1983). Phytoplankton and macrophytes have a high capability to become enriched with heavy metals via absorption or adsorption routes (Revis et al. 1989; Chojnacka et al. 2005; Monteiro et al. 2011), and uptake of metals via roots and leaves, respectively (Guilizzoni 1991). Within fish species the elemental content depends on factors such as location, age, size, life history, habitat, feeding guild and exposure route (Andres et al. 2000; Farkas et al. 2003; Vijver et al. 2004; Merciai et al. 2014; Van Ael et al. 2017; Ahmed et al. 2019). Via dietary exposure, feeding on metal contaminated food can increase metal contents of the predator (McEneff et al. 2017). Metal contents in invertebrates, possible prey for fish, are dependent on similar factors (Goodyear and McNeill 1999; Besser et al. 2001; Vijver et al. 2004; Santoro et al. 2009). However, it is not the total metal content per se determining trophic transfer and predatory content, as it is also highly dependent on the bioavailability of the metal in the prey and the digestive capacity of the predator (e.g. Wallace and Lopez 1997; Vijver et al. 2004; Rainbow and Smith 2010). To prevent intoxication, many organisms can also regulate the accumulation of metals (e.g. Vijver et al. 2004).

In general there are some strategies that organisms use to deal with excess metals. Essential metals can be regulated by limiting metal uptake, active excretion and/or storage in an inert form (Vijver et al. 2004). Non-essential metals mostly will be excreted from the excess pool or stored internally. The internal metals can subsequently be present in metabolically available (or bioavailable) form and/or in detoxified form (Rainbow 2002). Stored forms can be so-called metal-rich granules (MRG) and metallothioneins (MT) (Vijver et al. 2004). The present form of the metals is not only important to the organism but also for the transfer to higher trophic levels.

Filter feeders such as dreissenids, typically bio-accumulating organisms (Naimo 1995; Matthews et al. 2014), regulate their metal bioavailability for example. This is done by detoxification by MT (Marie et al. 2006), storage of metals in MRG or byssus threads (van der Velde et al. 1992; Marigómez et al. 2002; Vijver et al. 2004) or biodeposition (excretion) via (pseudo)faeces (Klerks et al. 1997; Marigómez et al. 2002). By their filtering capacities, they accumulate contaminants which can be transferred further in the food web (Naimo 1995; Al-Aasm et al. 1998; Camusso et al. 2001). Especially the Ponto-Caspian invasive false mussels (Dreissenidae) filter large quantities of water, changing the pelagic–benthic coupling by contributing to the benthic-littoral energy pathway (Higgins and Vander Zanden 2010). Via elevated metal contents in their biodeposited (pseudo)faeces (Klerks et al. 1997) metals can be incorporated in the benthic community (Goodyear and McNeill 1999). Benthivorous fish, such as round gobies (*Neogobius melanostomus*), that have a diet which includes mussels and other benthic macro-invertebrates, may accumulate the contaminants and transfer them to higher trophic levels when being eaten (Rutzke et al. 2000; Hogan et al. 2007; Subotić et al. 2013; McEneff et al. 2017; Verstijnen et al. 2019).

Metals will not necessarily biomagnify in higher trophic levels. Biodilution regularly occurs. Copper, cadmium, lead, and zinc are examples of heavy metals that are found to biomagnify but also biodilute in aquatic food webs (e.g. Chen et al. 2000; Gray 2002; Croteau et al. 2005; Watanabe et al. 2008; Schneider et al. 2018). Analysing the food web structure can help to unravel paths of biomagnification and biodilution.

Stable isotope analysis is often used to unravel food web structures and is helpful with determining trophic levels and possible interactions (e.g. Alcorlo and Baltanas 2013; Verstijnen et al. 2019; Balzani et al. 2021). It therefore can be used to assess biomagnification or biodilution of metals as well. On the other hand, as (heavy) metals are being transferred throughout the food web, metal analyses of the organisms potentially contribute to a better insight in the food web’s structure and trophic linkages.

A previous study unravelled the food web structure of three artificial reservoirs (Biesbosch, the Netherlands) based on carbon and nitrogen stable isotopes (Verstijnen et al. 2019). The reservoirs receive water from the River Meuse and are used for water storage and quality improvement (i.a. sedimentation of suspended solids, lowering of organic substances, natural purification) of the Meuse water. The water from the reservoirs is eventually used for drinking water production. The Meuse and its floodplains have been impacted by several anthropogenic activities in the past, which have led to enhanced metal concentrations in the surface water and sediments (Vink et al. 1999; Middelkoop 2000; Hobbelen et al. 2004). Although contamination has decreased since the 1970s (Middelkoop 2000), heavy metals are still present in the riverine system.

In this study, we examine how (heavy) metals and other elements transfer through the food web of the Biesbosch reservoirs (using the isotopic signatures); and if analysing metals in various (groups of) organisms in the food web could contribute to the understanding of the food web’s trophic levels and linkages. We have studied twenty elements but will focus on two essential (heavy) metals (Zn and Cu) and two non-essential (heavy) metals (Pb and Cd). The accumulation of heavy metals is often studied in aquatic food webs and in many studies no biomagnification of Zn, Cu, Cd and Pb is found. Therefore we also focus on two metabolically essential non-metals (P and Se), which are studied less frequently. By combining these element contents with isotopic signatures we might be able to gain a better insight in the transfer of elements in the food web. Furthermore by taking all twenty measured elements into account we also search for more possible linkages within this system.

The specific research parts of this study are: (A) determine elemental contents in (important) organisms in the reservoir food web, e.g. primary producers, zooplankton, fish, mussels and some other macro-invertebrate species, (B) determine if and how these elemental contents are correlated with trophic level and carbon source within the food web, (C) elucidate if possible trophic linkages between species can be based on contents of heavy metal and other elements and (D) get insight in possible biomagnification in the highest trophic levels (fish species). For the present study, heavy metals and other elements were analysed in the same tissue samples as the stable isotopes (Verstijnen et al. 2019).

## Methods

### Study Location

**Fig. 1:**
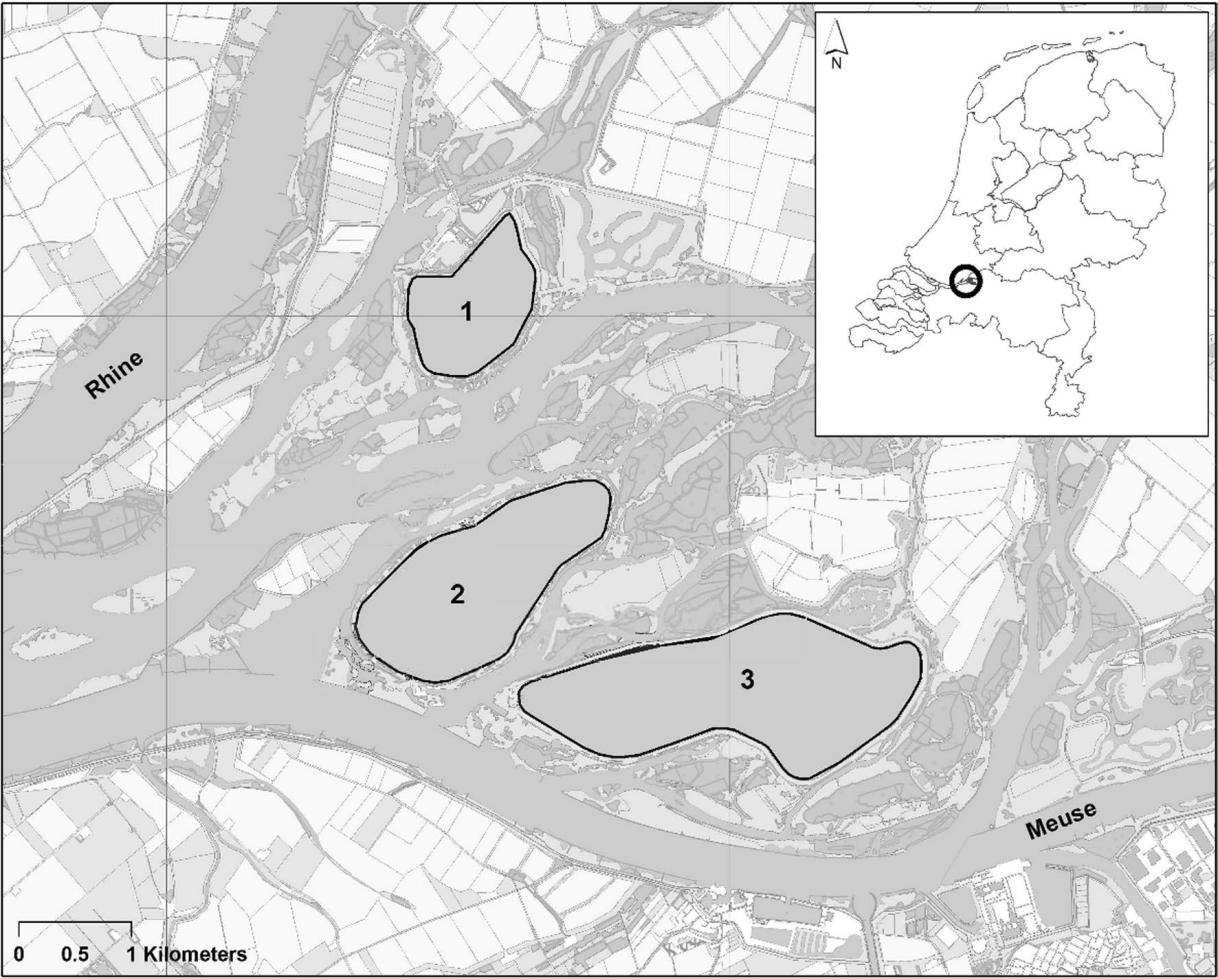
Locations of the three reservoirs in the Brabantse Biesbosch: (1) Petrusplaat, (2) Honderd en Dertig and (3) De Gijster (Verstijnen et al. 2019, © under CC BY 4.0). Water flows in South-North direction from River Meuse to (1) Petrusplaat

In the Brabantse Biesbosch (51° 44″ N, 4° 46″ E), the Netherlands, three reservoirs were constructed in the 1970s to abstract and store river Meuse water for drinking water processing (Fig. 1). De Gijster (area 320 ha, average depth 12 m, maximum depth 27 m), Honderd en Dertig (area 219 ha, average depth 16 m, maximum depth 31 m) and Petrusplaat (area 105 ha, average depth 12 m, maximum depth 15 m) were constructed for water quality improvement of Meuse water by natural purification processes. The reservoirs are interconnected belowground and their high volume storage allows for selective intake of river water. The reservoirs have relatively steep banks and hardly any littoral zone is present. The water is oxygenated by air injection units from April–October to prevent stratification and algal blooms in summertime. Verstijnen et al. (2019) presented more details on the reservoirs and their water quality. Annual average concentrations of various metals of the inflowing Meuse water and water in the outlet of the reservoir system are presented in Table SI1 and sediment data of Petrusplaat in Table SI2.

### Sampling

Various groups of organisms were collected in 2016 (May, June, August), 2017 (June, July) and 2018 (May, June). Samples from 2016 and 2017 have been analysed on stable isotopes by Verstijnen et al. (2019) and the same material was used to conduct elemental analysis. Samples were collected from various locations in the reservoirs and include cyanobacteria, seston (including phytoplankton), macrophytes, zooplankton (diverse species), fish, crayfish, sponges and other macro-invertebrates, including quagga mussels (*Dreissena rostriformis bugensis*). Detritus from mussel banks, a result of filtration of particles and subsequent release of faeces and pseudofaeces by mussels and of other settled particles from de water column, was collected as well. Most of the algae, cyanobacteria, sponges and macrophytes were manually collected from the shore or shallow banks, by using nets or by snorkelling/scuba diving (≤ 6 m). Zooplankton was collected with plankton nets (several mesh sizes). By using seine and multimesh gill nets and trawls, fish of various species were collected. Quagga mussels were collected with an Eckman grab sampler or manually during snorkelling. In most cases, macro-invertebrates and other samples were manually taken out of collected sediments at various depths. See Table SI3 and Verstijnen et al. (2019) for more detailed information on the sampling methods.

### Sample Processing

Samples were identified, if possible to species level. Fish (standard length and fresh weight) and quagga mussels (shell length and fresh soft tissue weight) were measured. Hereafter, samples were stored in the freezer (− 20 °C), later on transported to the laboratory for further analysis, rinsed with demineralized water and subsequently stored at − 80 °C. Subsequently, samples were freeze-dried for at least 48 h. Of fish, only muscle tissue without skin was taken from the flank of the fish (above the lateral lines and beneath the dorsal fin). Muscle tissue represents the highest biomass and longer-term assimilation, and can be directly related to the measured stable isotopes in the muscles (Verstijnen et al. 2019). Shells of mussels were removed and only the soft body tissue was used for analysis. For crayfish, the soft muscle tissue collected from the claws was used. Per macrophyte specimen, a mixture of stem and leaf material was used as a pooled sample. For other organisms, such as zooplankton and macro-invertebrates, whole individuals were used, where one sample consisted of multiple organisms to be large enough for analysis. After freeze-drying, all samples were ground to a fine powder in a bullet grinder (Retsch, Aartselaar, Belgium). The data of all three reservoirs were lumped together for all further analyses, as the reservoirs are interconnected. Physicochemical water quality variables of the reservoirs are within the same order of magnitude (Verstijnen et al. 2019).

A homogenised portion of dried material per sample was weighted into a Teflon container and digested with 4 ml HNO_3_ (65%; VWR international, Netherlands) and 1 ml H_2_O_2_ (30%; Sigma Aldrich, Merck Darmstadt, Germany), using a microwave oven (Ethos Easy, Milestone Inc., Sorisole, Italy). Digestates were diluted to 100 ml with Milli-Q water and analysed for Al, Ca, Fe, K, Mg, Mn, Na, P, S, Si and Sr using an Inductively Coupled Plasma Spectrometer (ICP-Optical emission spectrometer (ICP-OES), iCAP 6300, Thermo Fisher Scientific, Bremen, Germany), equipped with a concentric nebulizer chamber and a V groove nebulizer and radial plasma observation. The RF power was set to 1150 W and nebulizer gas flow of 0.7 l/min. Other (e.g. heavy metal) contents (As, Cd, Co, Cr, Cu, Ni, Pb, Se and Zn) were determined with ICP-Mass Spectrometry (ICP-MS, X-series and iCAP T-RQ, Thermo Fisher Scientific, Bremen Germany), with axial plasma observation, 1200 W plasma power and a seaspray nebulizer combined with a concentric nebulizer chamber. Instrumental limits of detection are given in Table SI4. For the ICP measurements calibration standard mixes were used from Certipur (Merck, Darmstadt, Germany). For the quality control individual standards were used of the same manufacturers. Every 12 samples a quality control standard was measured and checked for a maximum difference of 10%. For the ICP-MS Scandium was added automatically as internal standard and was checked for recovery. Recoveries from internal standards ranged 90–112%. Repeated analyses did not reveal differences greater than 10%. Elemental contents were measured in ppb. The data were converted to µg/g dry weight (DW) of the sample.

### Data Analyses

The sampled species/groups were classified according to their main feeding guild based on their food sources. The main feeding guilds were determined based on expert knowledge, stomach contents of fish (Evides, unpublished data), FishBase (Froese and Pauly 2021) and literature (Elliott and Dewailly 1995; Aarts and Nienhuis 2003; Zambrano et al. 2006; Platvoet et al. 2009; Kornis et al. 2012; Vojkovská et al. 2014; Schachtl 2019; Verstijnen et al. 2019). Fish species were divided into size classes, as was done earlier by Verstijnen et al. (2019), based on length and dietary information.

Biomagnification factors (BMF) or Trophic Transfer factors (TFF) (e.g. DeForest et al. 2007; Presser and Luoma 2010) were calculated for predator–prey relations of three fish species [ruffe (*Gymnocephalus cernua*), roach (*Rutilus rutilus*) and round goby (*Neogobius melanostomus*); based on their muscle tissue]. BMF or TFF is the ratio of the metal content in the predator to the content in its dietary items. Per fish species we took the two main dietary items into account, as established earlier by Verstijnen et al. (2019).

$${\text{BMF or TFF}} = {{{\text{Metal}}_{{{\text{predator}}}} } \mathord{\left/ {\vphantom {{{\text{Metal}}_{{{\text{predator}}}} } {\sum_{{{\text{prey}}}} \left( {p_{{{\text{prey}}}} *{\text{ Metal}}_{{{\text{prey}}}} } \right)}}} \right. \kern-0pt} {\sum_{{{\text{prey}}}} \left( {p_{{{\text{prey}}}} *{\text{ Metal}}_{{{\text{prey}}}} } \right)}},$$

where Metal_predator_ and Metal_Prey_ are the contents (µg/g DW) of the selected metals in the predator and prey, respectively. P_prey_ is the proportion of each prey in the predator’s diet (Balzani et al. 2021).

All analyses and graphics were done or created using R statistics (version 3.6.1). A heatmap involving all twenty measured elements was created to investigate correlations between elements (R package: corrplot). To check for similarities between species/groups a dendrogram was made based on all twenty elements (R package: ggdendro). In several samples, the measured Cd contents were lower than the detection limit. For analyses and visual presentation of the data, the lowest measured content was therefore added to all Cd contents (0.08110 µg/g). Graphs are thus shown scaled for Cd (where applicable therefore being referred to as ‘adjusted’ contents). The same was done for As (0.08621 µg/g). The elements of P, Cd, Cu, Pb, Se and Zn were further investigated. Cu, Cd, Pb and Zn are frequently studied metals (Goodyear and McNeill 1999), as elevated levels might be toxic to organisms. Zn and Cu are essential trace metals; whereas, Cd and Pb are in general considered non-essential (e.g. Rainbow 2002; Hejna et al. 2018). P is an essential macronutrient; whereas, Se is an essential trace nutrient. We therefore chose these elements to focus on as they (amongst others) can be considered ecologically relevant. Pearson correlation was determined on the log-transformed means per group for these elements versus δ^15^N and δ^13^C. Seston and detritus were excluded in correlations with δ^15^N, as these comprised partly non-living material. A nonlinear regression analysis was performed on fish length versus the six elements. When data were not normally distributed, log transformations were executed. An ANOVA on ranks was executed to compare means between groups.

## Results

### Food Web Elemental Composition

**Fig. 2:**
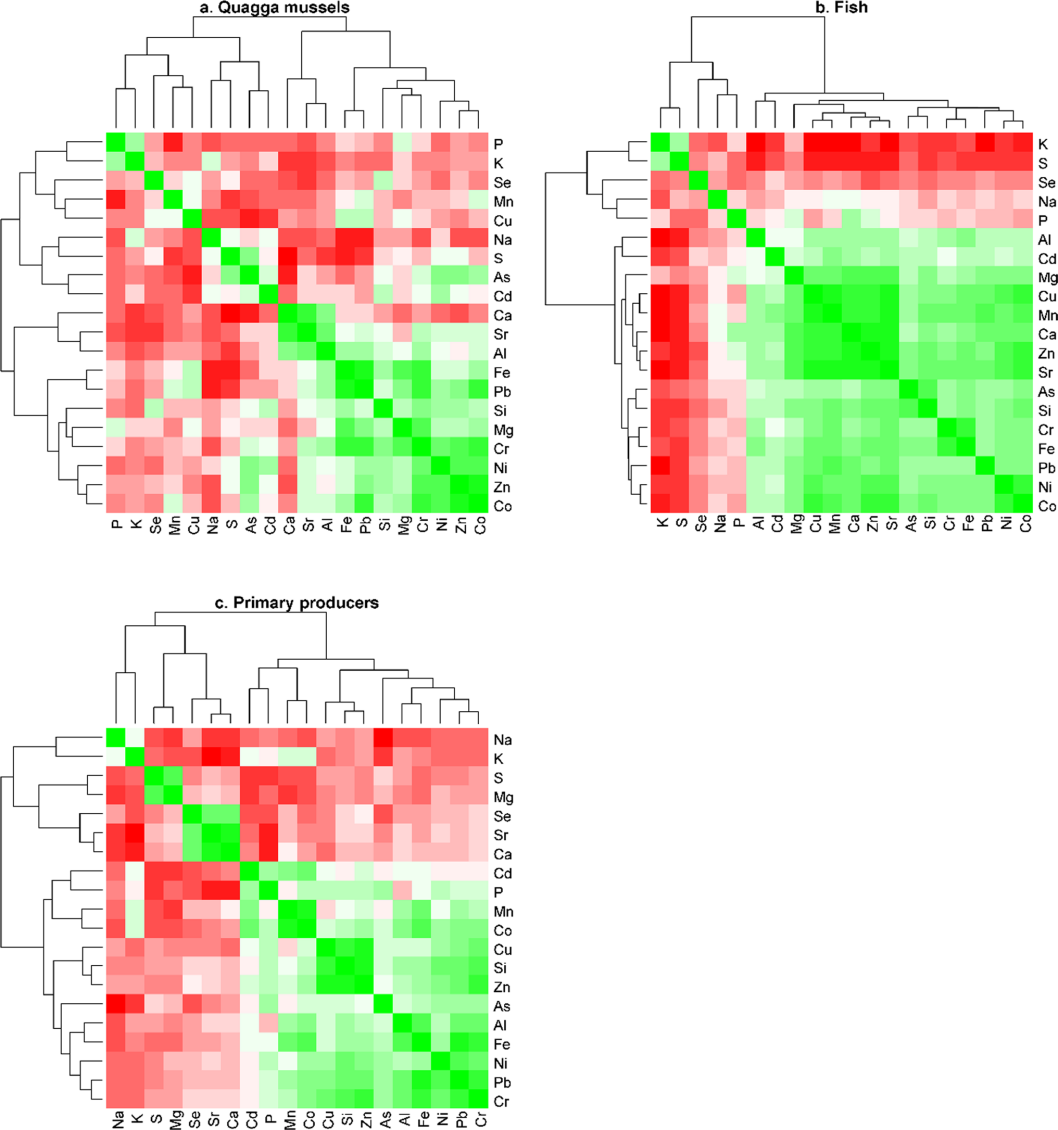
Heatmaps of the correlations between elements, separately for quagga mussels, fish and primary producers. Red = negative correlation, green = positive correlation. The brighter or more intense the colour, the stronger the correlation. Bright red: *r* = − 1. Bright green: *r* = 1

Many elements were negatively or positively correlated with each other. Based on a correlation matrix, elements that showed similar correlations with the other elements were clustered together. The resulting ‘heatmap’ of these correlation matrix, including all samples, is shown in Figure SI1. Per species group the strength of correlations did differ (Fig. 2). Where, for example, potassium (K) and sulphur (S) had a strong negative correlation with almost all other elements in fishes (Fig. 2b), these correlations were less strong for primary producers (plants and algae including phytoplankton) and quagga mussels (Fig. 2a, c). Along the different clusters, six ecologically relevant elements were depicted for further analyses: phosphorus (P), cadmium (Cd), copper (Cu), lead (Pb), selenium (Se) and zinc (Zn) (see also “Methods” section: data analyses). In the reservoirs, the concentrations of these metals were lower in the outflow compared to the river water inflow (Table SI1).

**Fig. 3:**
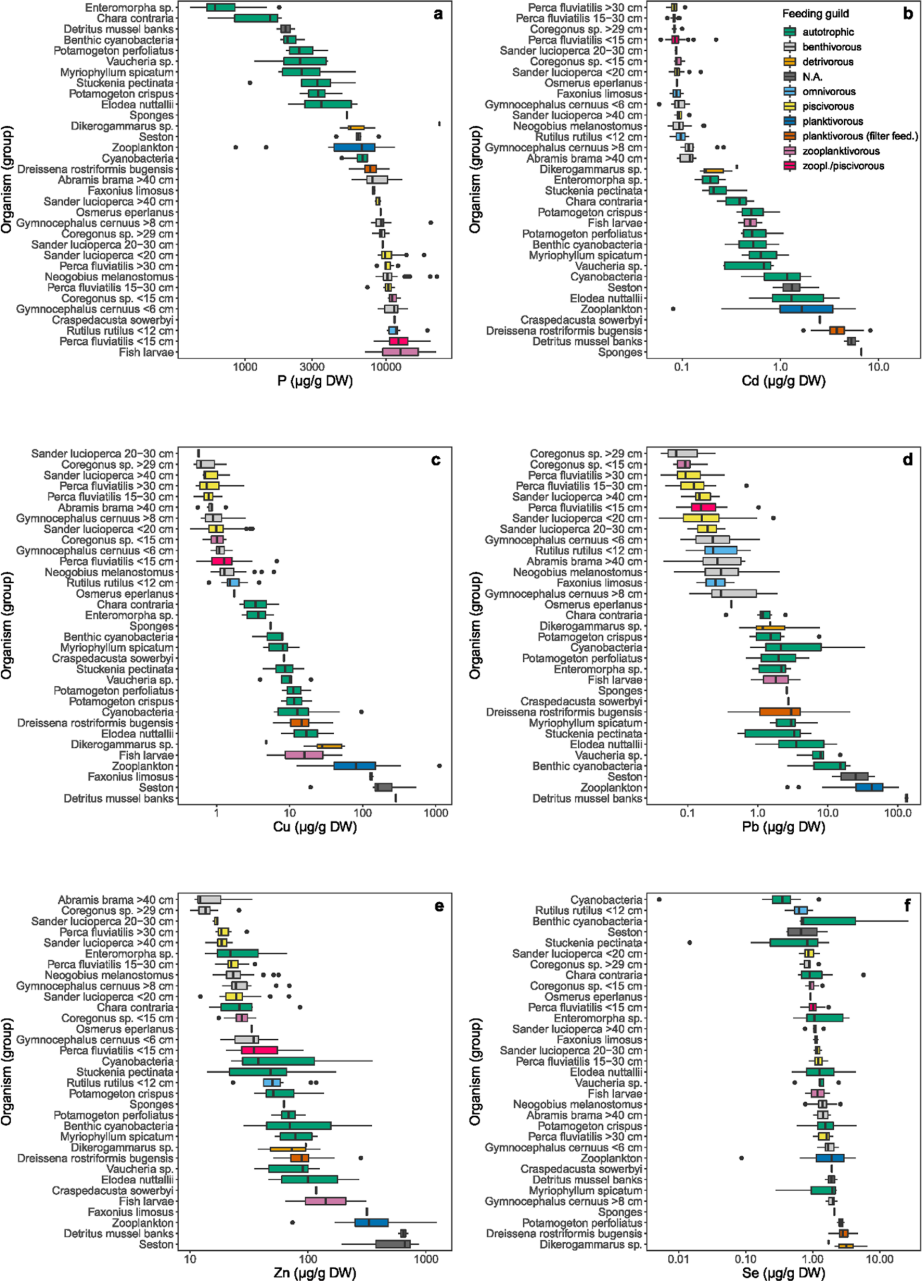
Boxplots showing i.a. the 25th (left box), 50th (median) and 75th (right box) percentile of the selected relevant elements, depicted from the different clusters (Fig. 2), sorted on ascending median values (µg/g dry weight), grouped per organism/taxon and coloured by feeding guild. **a** phosphorus, **b** cadmium (based on the adjusted values), **c** copper, **d** lead, **e** zinc and **f** selenium. *N.A.* no applicable feeding guild (mussel bank detritus and seston)

Related species or species belonging to the same feeding guild often had similar elemental contents (Fig. 3, Table SI5). For P, the lowest contents were measured in mussel bank detritus and autotrophic organisms (plants/macro-algae) such as *Potamogeton* species (median range 614–3480 µg/g DW). Highest P contents were measured in the fish muscle tissue and fish larvae (median range 8028–14869 µg/g DW) and intermediate values in macro-invertebrates.

A different trend was found for Cd, with the lowest contents measured in the fish species and crayfish (median mostly < 0.1 µg/g DW). The highest contents were found merely in filter feeding and planktivorous animals. *Dikerogammarus* (*D. villosus* and *D. haemobaphes*) had intermediate levels for Cd (median based on the adjusted data: 0.17 µg/g DW, see methods).

As for Cd, the lowest Cu and Pb contents were found in the fish (median ≤ 1.76 µg/g DW for Cu and ≤ 0.42 µg/g DW for Pb). Intermediate levels were found in autotrophic organisms and in sponges, jellyfish and quagga mussels, ranging for Cu from 3.57 µg/g DW (median *Chara contraria*) to 17.02 µg/g DW (median *Elodea nuttallii*). For Pb, *Dikerogammarus* sp. also contained intermediate levels (median 1.21 µg/g DW). The highest contents were found in (zoo)plankton/seston and mussel bank detritus (Fig. 3c, d) and for Cu also in crayfish (*Faxonius limosus*).

Likewise, Zn contents were the lowest in fish muscle tissue (Fig. 3e). Contrary to Cd, Cu and Pb the Zn contents of macro algae *Enteromorpha* and *Chara contraria* were also within the range of those of the fish. *Potamogeton* species had Zn contents similar to roach (*Rutilus rutilus*).

Se did not show clear trends divided over feeding guilds (Fig. 3f). The lowest contents were found in cyanobacteria and the highest contents in *Dikerogammarus*.

Within the fish, a distinguishable trend/grouping of benthivorous versus piscivorous or omnivorous species for the measured elements could not be demonstrated. Though benthic/demersal fish (*Abramis brama, N. melanostomus, G. cernua*) seem to have similar contents (Fig. 3, Table SI5), which were in general higher than in pelagic fish.

### Food Web Trophy and Interaction

**Fig. 4:**
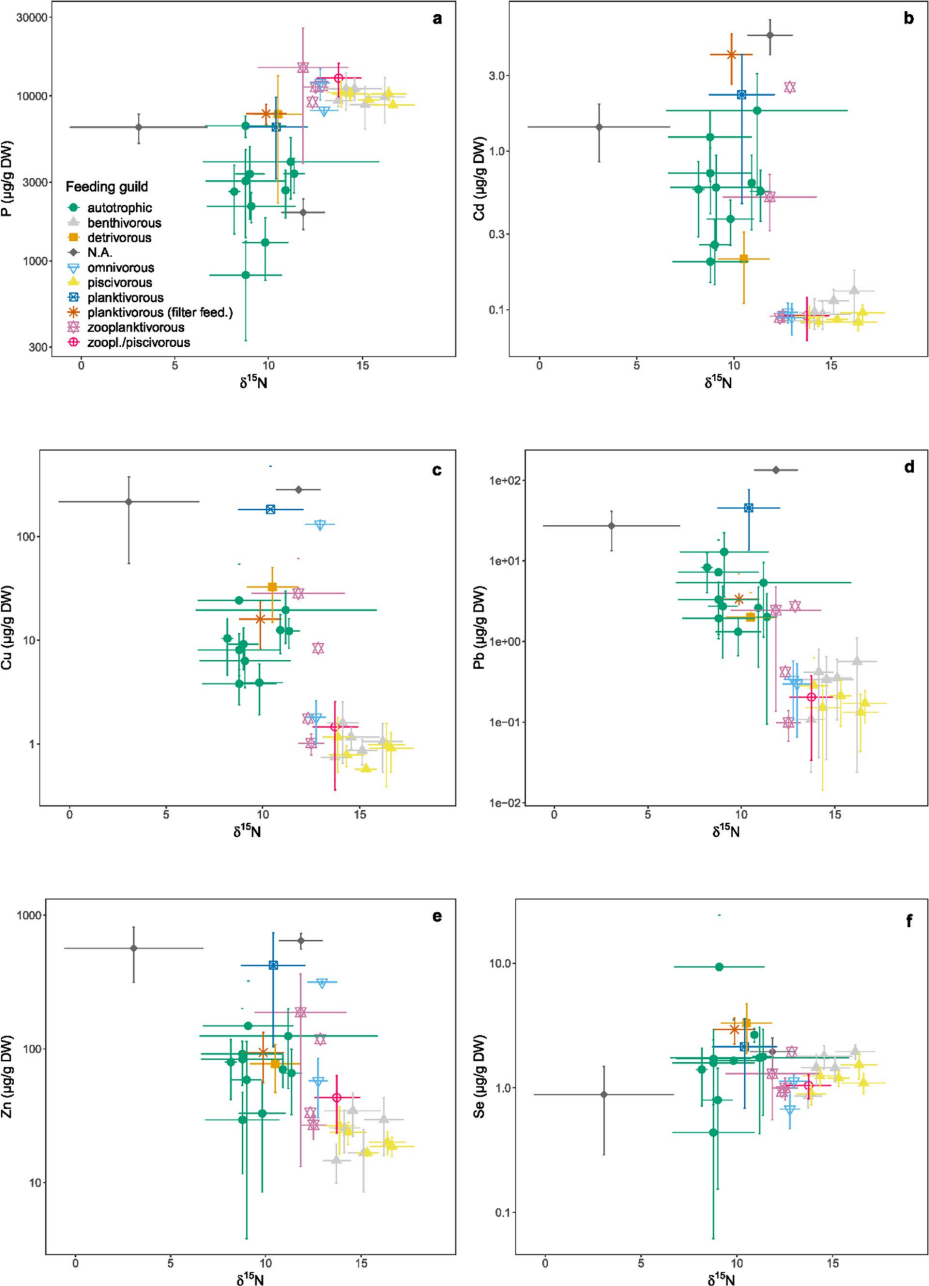
Average elemental contents (µg/g dry weight ± standard deviation; on a log-scale) of the selected relevant elements (depicted from the different clusters in Fig. 2) versus δ^15^N (stable isotope ratios from Verstijnen et al. 2019), grouped per organism/taxon and coloured by feeding guild. **a** phosphorus, **b** cadmium (based on the adjusted values), **c** copper, **d** lead, **e** zinc and **f** selenium. *N.A.* no applicable feeding guild (mussel bank detritus and seston)

When plotting the metal contents against trophic level, measured as δ^15^N (Verstijnen et al. 2019), the heavy metals Cu, Cd, Pb and Zn were significantly (*p* < 0.05) negatively correlated (Fig. 4; *r* = − 0.65, − 0.72, − 0.79, − 0.57, respectively). Lower contents were found in organisms with higher δ^15^N values. Higher trophic positions were occupied by fish species; while, the lower trophic positions were occupied by (phyto)plankton followed by cyanobacteria and macrophytes. The average P contents correlated positively with δ^15^N (Fig. 4; *r* = 0.69, *p* < 0.05) and no correlation was found between Se and δ^15^N (*r* = − 0.15, *p* = 0.43).

**Fig. 5:**
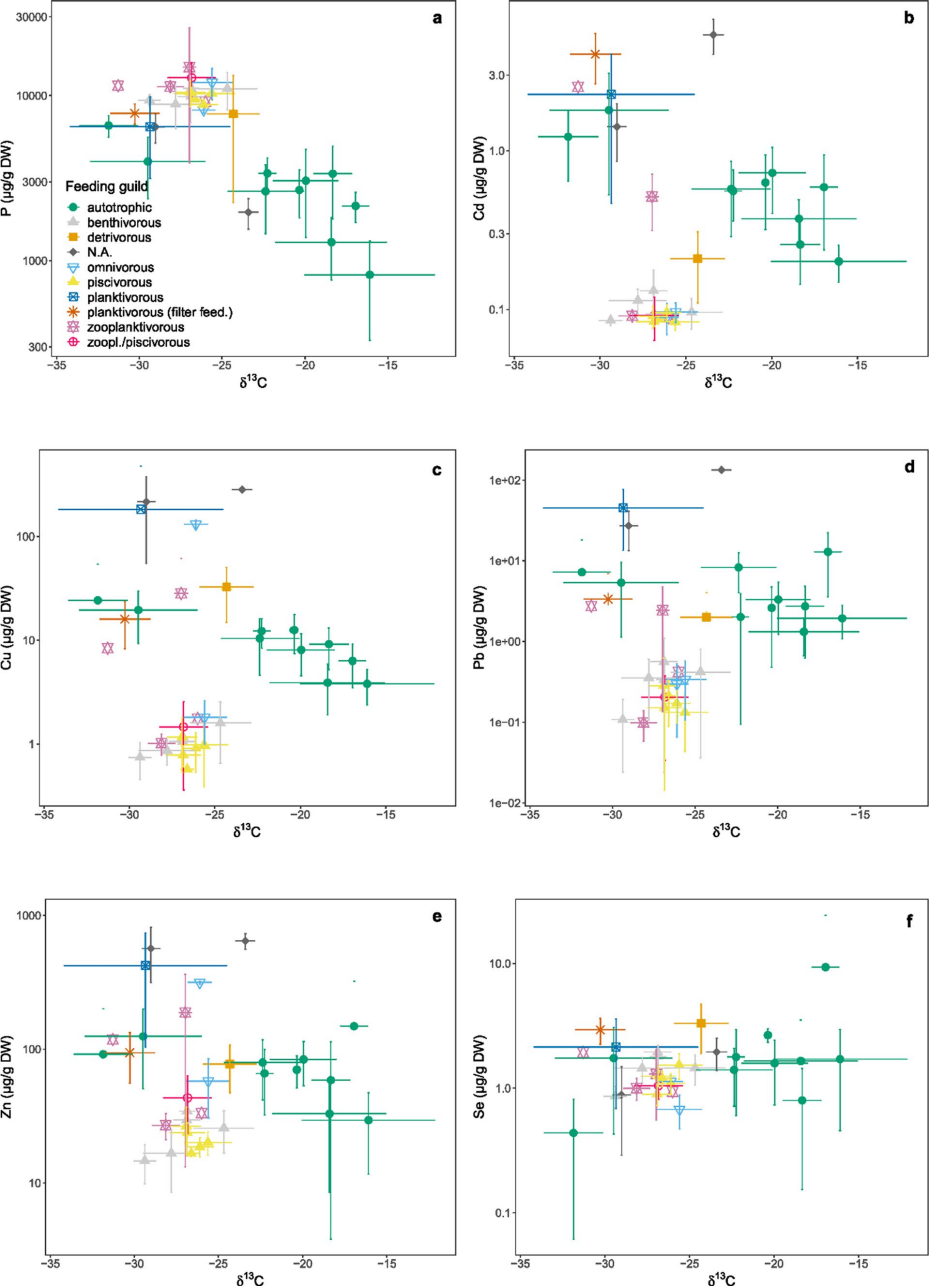
Average elemental contents (µg/g dry weight ± standard deviation; on a log-scale) of the selected relevant elements (depicted from the different clusters in Fig. 2) versus δ^13^C (stable isotope ratios from Verstijnen et al. 2019), grouped per organism/taxon and coloured by feeding guild. **a** phosphorus, **b** cadmium (based on the adjusted values), **c** copper, **d** lead, **e** zinc and **f** selenium. *N.A.* no applicable feeding guild (mussel bank detritus and seston)

Where δ^15^N is an indicator of the trophic level in a food web, δ^13^C gives an indication of the carbon source. Correlations between the elemental contents and the averaged δ^13^C were less evident as compared to δ^15^N. P contents correlated negatively with δ^13^C (*r* = − 0.76, *p* < 0.05), especially in the case of autotrophic organisms; the more enriched (less negative) the carbon signature, the lower the P-content (Fig. 5). Fish were the top predators in the food web with intermediate δ^13^C signatures (Verstijnen et al. 2019). Their muscle tissue had the lowest heavy metal contents and so they formed a distinguishable group in the plots of Cu, Cd, Pb and Zn contents versus δ^13^C. Without fish taken into account, there was a significant (*p* < 0.05) negative correlation between Cu, Cd, and Zn contents with δ^13^C (*r* = − 0.52, − 0.57, − 0.49, respectively). There was no such correlation for Pb (Fig. 5).

The heavy metal contents not only differed by feeding guild and trophic level, as is visible in Figs. 3, 4 and 5, but also by organic source of the food web (macrophyte, phytoplankton or a mix, based on δ^13^C) (Figure SI2). Highest Cd and Zn contents occurred in the phytoplankton-based carbon pathway (Figure SI2b,e: *H* = 264, 170 respectively, *df* = 2, *p* < 0.001). To a lesser extent this was also true for Cu and Pb. The lowest contents were found in the mixed source, which corresponds with fish species. The macrophyte-based groups often had intermediate heavy metal contents.

**Fig. 6:**
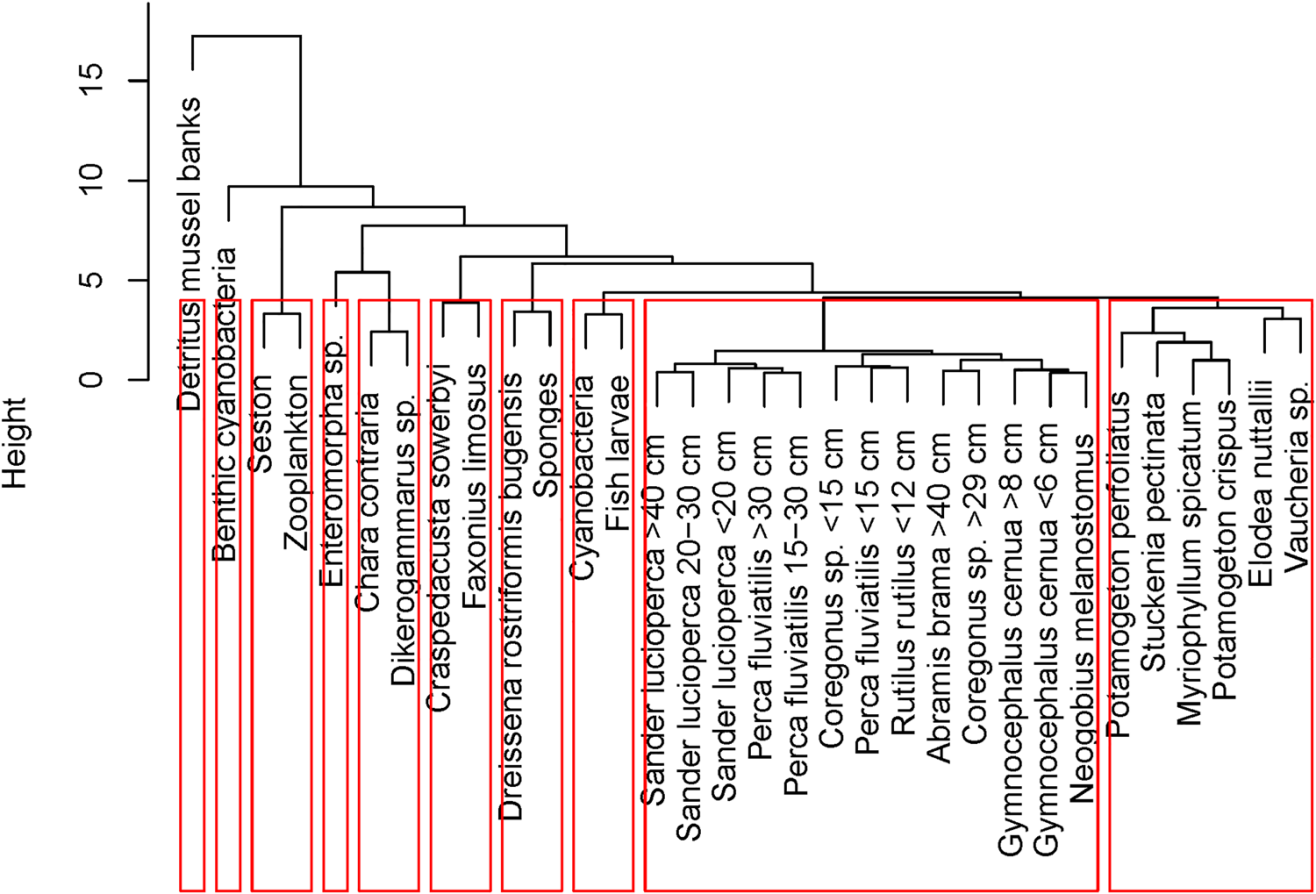
Dendrogram based upon elemental contents. The red boxes indicate clusters divided over ten groups. The height of the branches indicates the degree of similarity between each other

The similarities between organisms based upon their elemental/metal contents (Figs. 3, 4 and 5) were further analysed via a cluster analysis (Fig. 6). When dividing the various organisms over ten groups, all fish species clustered together. Likewise, plankton (zooplankton and seston including phytoplankton) clustered together. Benthic cyanobacteria became closely clustered to the plankton group but formed a separate group. Nearly all macroalgae and macrophytes clustered together. *Chara contraria* was however clustered with the gammarid *Dikerogammarus* sp. Carnivorous jellyfish and omnivorous crayfish clustered together, just as the filter feeding quagga mussels and freshwater sponges. The green macroalgae *Enteromorpha* was closely related to the *Chara* and *Dikerogammarus* sp. cluster.

**Fig. 7:**
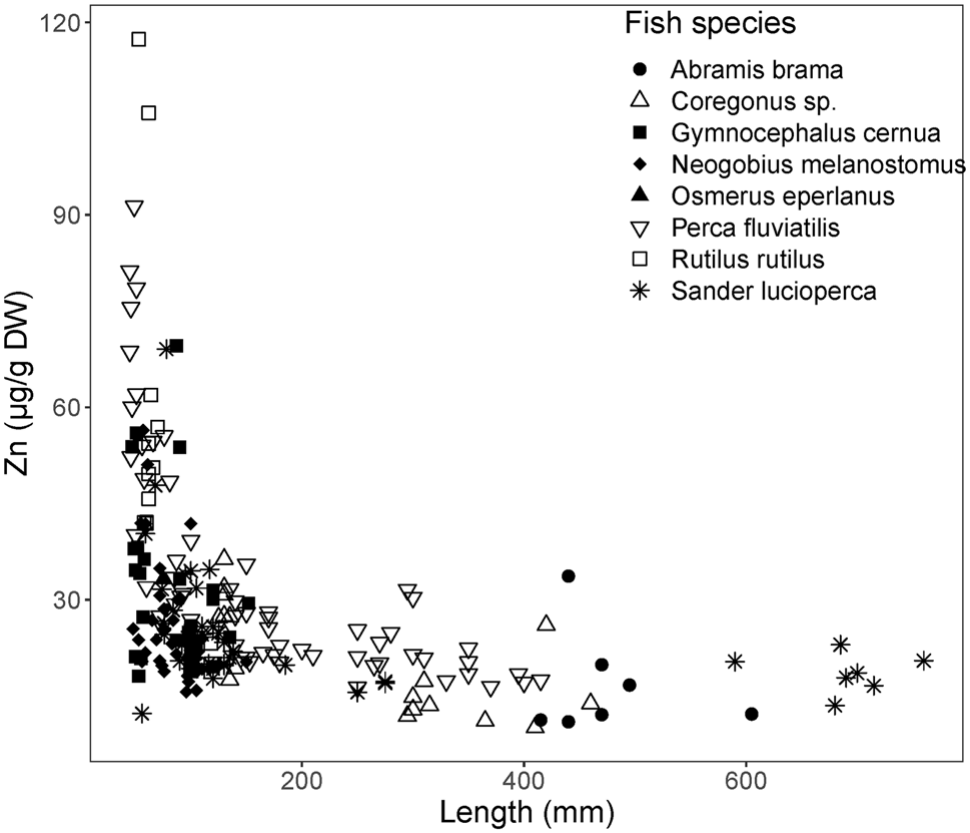
Zinc contents (µg/g dry weight) related to fish length (mm) of the studied species

A clear clustering of taxonomic groups based on their feeding guild was not observed (Figs. 3 and 6), though the elemental signature of most benthivorous fish seemed to differ a little from those of piscivorous fish (Fig. 6). We did observe a nonlinear relation between elemental contents (except for Cd and Se) and fish length (Fig. 7; Zn: *R*^2^ = 0.46, *DF* = 211, *p* < 0.001) when taking all species into account. Larger fish often had lower metal contents than the smallest specimen of the same species (Fig. 7 and Figure SI3b). This suggests that at growth there was no bioaccumulation but biodilution. Though there was not always a correlation between metal content and fish length for the separate fish species (Figure SI3).

Round goby and ruffe, which occurred in a similar niche and habitat (Jůza et al. 2018; Verstijnen et al. 2019), did have similar elemental signatures (Fig. 6) and contents (Table SI5). Also roach had similar contents concerning some elements. However, Se was lower and Zn higher compared to ruffe and round goby. Within ruffe, round goby and roach, three species in possible competition (Verstijnen et al. 2019), there was no biomagnification of heavy metals in their muscle tissue (Table 1). The BMF calculations (Table 1) were based on several main dietary items of these fish (*D. rostriformis bugensis*, *Dikerogammarus* sp. and/or *Stuckenia pectinata*), as specified in Verstijnen et al. (2019). For the heavy metals and selenium, biodilution (BMF < 1) was observed (Table 1). Biomagnification (BMF > 1) was found for phosphorus.

**Table 1:** Biomagnification factor (BMF) of P, Cd, Cu, Pb, Se and Zn for ruffe (*G. cernua*), round goby (*N. melanostomus*) and roach (*R. rutilus*) based on a combination of diets (*D. rostriformis bugensis*, *Dikerogammarus* sp. and/or *S. pectinata*)

	P	Cd	Cu	Pb	Se	Zn
Fish species						
*G. cernua*	1.33	0.04	0.05	0.16	0.62	0.35
*N. melanostomus*	1.42	0.06	0.06	0.17	0.46	0.31
*R. rutilus*	1.99	0.04	0.14	0.11	0.32	0.72

## Discussion

### Heavy Metals, Se and P in the Food Web

There are differences in heavy metal content between the sampled organisms in the reservoir food web. Heavy metal contents in the piscivorous, benthivorous and omnivorous animals (mainly fish), were clearly lower than in autotrophic organisms and the filter feeders (Quagga mussels and sponges). Quagga mussel bank detritus contained the highest amounts of heavy metals. Phosphorus contents showed an opposite trend and no obvious pattern emerged for selenium.

The absence of clear patterns for Se between organisms or feeding guilds can imply that it is not present in excess amounts. In excess amounts Se can be toxic, especially for fish (Chapman et al. 2010). It is an essential trace element for organisms though and may play a role in the detoxification of some metals (Ikemoto et al. 2004).

P is essential for all organisms to grow. In fish it is an important part of their bone and scales, but accumulates in soft (e.g. muscle) tissue as well (Lall and Lewis-McCrea 2007). This might explain the high P contents found in fish muscle tissue.

Metabolic and physiological characteristics play an important role in the observed differences in metal contents (Schneider et al. 2018) between the studied organisms. Our results revealed higher contents of metals in aquatic macrophytes and seston (suspended particles including phytoplankton). This can be related to the metal uptake via roots and leaves (macrophytes), and adsorption (phytoplankton) (e.g. Revis et al. 1989; Guilizonni 1991). Nevertheless, we found a difference between the rooted *E. nuttallii* and *Potamogeton* species. *Elodea* had on average higher contents of Zn, Cd, Pb and Cu. This might be related to differences in biomass, higher leaf uptake and surface:volume ratio by *Elodea* and/or its higher water content (low dry weight:fresh weight ratio) as discussed by Fritioff et al. (2005). δ^13^C values also differed between these species (Verstijnen et al. 2019), which reflects different uptake characteristics. Furthermore, *Chara contraria* had similar δ^13^C and δ^15^N values as *Potamogeton* species, though their elemental content is slightly different (Fig. 3), with often lower metal contents. Where *Potamogeton* species root and can take up metals from the sediment, *Chara* has rhizoids which may be less well adapted to take up metals from the sediment. Metal analysis can thus reveal differences between algal and rooted autotrophs.

Different species of zooplankton might also have different metal accumulation (Zauke et al. 1996). In our study, zooplankton was however measured as a mixture of species present at that moment. With this sampling strategy the relative abundance of a certain species was taken into account. Possible selective uptake of certain zooplankton species by predators could however not be determined, although this might influence transfer to higher trophic levels.

For organisms higher in the food web, the potentially toxic heavy metals will be either excreted or internally detoxified in order to prevent intoxication. Crayfish for example had high contents of (essential) Zn and Cu whereas contents of (non-essential) Pb or Cd were relatively low. This might be due to the detoxification of the non-essential metals by MT as discussed by Alcorlo and Baltanas (2013). The filter feeding quagga mussels accumulate high levels of metals (e.g. Naimo 1995; Matthews et al. 2014). Their filtering potentially contributes to lower surface water concentrations (Rutzke et al. 2000) in the Biesbosch reservoirs (Table SI1). Although the mussels regulate bioavailability via storage and excretion (Marigómez et al. 2002; Marie et al. 2006), the accumulation can cause internal metabolic changes (Louis et al. 2019). Fish can accumulate metals and experience effects as well. Non-essential metals as Cd for example can for an important part be retained in the kidneys (Kondera et al. 2014); whereas, an essential metal as Cu is regulated. Fish can thus actively redistribute and excrete heavy metals, often via hepatic or biliary excretion (e.g. Grosell et al. 1998; Le Croizier et al. 2019). The involved organs are likely to accumulate more metals; whereas, muscle tissue reflects diet origin and often contains lower content (Farkas et al. 2003; Subotić et al. 2015; Balzani et al. 2021). Because of these processes, the studied muscle tissue of the fish therefore probably had the lowest metal contents. In general sampling whole organisms, only soft body tissue (mussels), or only muscle tissue (fishes) could have influenced the patterns observed.

The relatively large differences in the contents of elements between species or other taxa, as found in this study, are observed in other studies as well (e.g. Rainbow 2002; Griboff et al. 2018; Balzani et al. 2021). Intraspecific comparison gives an idea of the relative levels. The studied quagga mussels contained Cd and Pb levels of 3.9 and 3.3 µg/g DW on average respectively, which are within the range found for quagga mussels in Lake Erie (Rutzke et al. 2000). Muscle tissue Zn contents for perch and pikeperch (*Sander lucioperca*) were overall lower in the Danube River (19 and 15 µg/g DW, respectively) (Subotić et al. 2015), compared to our results (see medians per size class in Table SI5; ranges perch and pikeperch 16–91 and 12–69 µg/g DW, respectively). In the native ruffe (*G. cernua*), Zn content was similar and Cd and Cu were higher in our study compared to ruffes found in the Danube River. Average Cd content in invasive round goby (*N. melanostomus*) was lower in our study and Cu was higher compared to a dead Vistula River channel (Wyrzykowska et al. 2012; based on a muscle dry weight:fresh weight ratio of 0.25, Creteanu et al. 2008). Thus some contents in fish were lower whilst some contents were higher compared to the literature data, indicating some influencing (environmental) factors, such as exposure route and location. Besides, whether or not a certain species accumulates and transfers metals, may be more related to trophic level and food web interactions rather than to taxonomic classification of the organisms (e.g. Quinn et al. 2003; Watanabe et al. 2008; Schneider et al. 2018).

### Food Web Trophy and Interaction

Cd, Pb, Cu and Zn contents of the samples correlate with food web trophic level in the reservoirs. They were negatively correlated with δ^15^N of the investigated species and some metals correlated with the carbon signature (δ^13^C) as well. For P, however, a positive correlation was found with δ^15^N; while, a clear negative correlation was found with δ^13^C. For Se no relationships between the contents and the isotopic signatures were found.

Similar negative correlations between metal contents and trophic level were found in other studies where several groups of aquatic organisms were studied. For example Besser et al. (2001) found a decrease in Zn and Pb across trophic linkages, but not for Cd and Cu. In the Arno River (Italy), Balzani et al. (2021) found negative correlations between trophic level and Cu as well as for Pb, but not for Cd and Zn. Similar results were obtained for a seagrass food web (Schneider et al. 2018). All four metal contents were however also dependent on food source in the seagrass food web, with organisms feeding on particulate organic matter and benthic microalgae having higher metal contents. This was also the case in our study, where ‘plankton-based’ organisms tended to have higher contents than ‘macrophyte-based’, i.a. for Cd.

However, in a creek system (Montana, USA) positive correlations were found between Zn and Cu content in benthic macro-invertebrates and trophic level, suggesting biomagnification in this food web (Chen et al. 2000). Also for Se a positive correlation in a food web was found by Kehrig et al. (2013), where we did not find any correlation. Furthermore, in the Scheldt Estuary no correlation was found between trophic level and metal content, as metal contents seemed to be more dependent on location and environmental concentrations (Van Ael et al. 2017). These results imply that metal content is often related to the trophic position, but this relationship and the metal contents can be dependent on (type of) species, type of metal and location (e.g. Quinn et al. 2003). In our study, trophic level and food source (for Cd and Zn) are indicative for the studied metal contents of an organism.

In our study, heavy metals are not biomagnified (biomagnification or trophic transfer) as is reflected by the negative correlations between the trophic levels and metal contents (Griboff et al. 2018). This negative correlation indicates biodilution. P, however, biomagnifies to higher trophic levels. The calculated BMF (or TFF) for ruffe, roach and round goby (Table 1), based on their diet, also reflected biodilution for heavy metals and biomagnification for P. This is in accordance with the fact that species higher up in the food chain, mainly fish, have mechanisms that regulate metal uptake, detoxification and excretion.

BMF was calculated based on knowledge on the diet of the predators, though not all possible diets were included. The diet proportions were derived from i.a. stable isotope analysis and literature on possible prey (Verstijnen et al. 2019). These proportions are determined under certain assumptions and are therefore not an exact value. This naturally feeds into the BMF. A change of diet (proportions) could increase or decrease the BMF, affecting our conclusions. Unless experimentally and extensively studied in the field, exact diet proportions are difficult to measure. Stable isotope analysis however gives an in situ display of the predator–prey relations (e.g. Kehrig et al. 2013).

When fish grow, their isotopic signature can change (Verstijnen et al. 2019) due to changing diets. In this study fish size and metal content in their muscle tissue is inversely related (Fig. 7). This is true for, e.g. perch and pikeperch. Especially very small fish tended to have (much) higher metal contents. Similar results have been found in other studies as well (e.g. Canli and Atli 2003; Merciai et al. 2014; Balzani et al. 2021). Smaller fish eat smaller dietary items with often higher metal content. In addition, fish growth may dilute muscle tissue content, as can also be the case for invertebrates (Karimi et al. 2010). Small fish have a higher relative metabolic activity and therefore consume relatively more food, as being discussed by Canli and Atli (2003). Because of the relationship between metal content and fish size it is important to take fish size into account when comparing intraspecific results. This we have done by dividing fish into size classes (Fig. 3).

Besides correlations with food web trophy, the elemental signature based on heavy metal and other elemental contents, can give insight in trophic linkages, as was demonstrated by cluster analysis (Fig. 6).

Mussel bank detritus had the highest metal contents of all investigated material, though in some cases similar to phytoplankton/seston. Benthic cyanobacteria and detritus did have a similar elemental signature (Fig. 6), indicating effective metal sorption (Baptista and Vasconcelos 2006). Additionally, we cannot exclude that some detritus particles were left attached to these cyanobacterial samples. The detritus consisted of i.a. the (pseudo)faeces of mussels and (most likely) other settled particles. Reeders and Bij de Vaate (1992) found slightly higher or similar contents in Meuse filtering zebra mussel (*Dreissena polymorpha*) pseudofaeces compared to the suspended matter. Detritus particles can be part of the suspended organic matter/seston, contributing to the metal content in the seston.

The seston including phytoplankton is typically a food source for the quagga mussels and zooplankton. Absorbed or adsorbed soluble and insoluble metals in phytoplankton can thereby be transferred to these higher trophic levels (Ng et al. 2005). Zooplankton metal contents can be highly correlated to phytoplankton, implying a direct feeding relationship (Tao et al. 2012) which was also found by stable isotope analysis (Verstijnen et al. 2019) and our cluster analysis (Fig. 6).

The metal contents in *Dikerogammarus* sp. (detritivores) are in general lower than in seston/phytoplankton and zooplankton, much lower than the detritus, but similar to or slightly lower than those in the mussels. Such detritivores may regulate their (non-) essential metal content (Maazouzi et al. 2008; Vellinger et al. 2013), which could prevent biomagnification. Goodyear and McNeill (1999) also did not find biomagnification of heavy metals in freshwater macro-invertebrates, in a meta-study. Maazouzi et al (2008) suggested that the regulation of certain metals as Cu by *D. villosus* could (indirectly) contribute in making it a successful invader (Maazouzi et al. 2008). In the reservoirs, the Cu concentrations were lower than those measured in the study of Maazoui et al. (2008) and lower than the LC50 for indigenous *Gammarus pulex* (Taylor et al. 1991). For Cd, Boets et al. (2012) found that *D. villosus* was actually more sensitive compared to indigenous species. In the Biesbosch reservoirs however, the Cd concentrations were well below the tested concentrations by Boets et al. (2012) (Table SI1). In this study we focussed on the dietary pathway rather than waterborne exposure routes. In the detritus, one of the *Dikerogammarus* food items, the metal content was high. Next to detritus, other possible diet sources are oligochaetes and other crustaceans (Bacela-Spychalska and van der Velde 2013), which were not measured in our study. These possibly had lower metal content than detritus and might have also affected the *Dikerogammarus* metal content.

*Dikerogammarus* sp. is an important dietary item for the native ruffe and the invasive round goby (Emde et al. 2014; Verstijnen et al. 2019) but no biomagnification of metals is demonstrated in fish muscle tissues of both species (BMF < 1; Table 1). Their BMFs are similar and they cluster together based on their elemental contents (Fig. 6). A likely competition between these species in the Biesbosch reservoirs has already been established by Jůza et al. (2018) and Verstijnen et al. (2019). The elemental signature and BMFs may imply similar metal accumulation strategies (storage and excretion) and trophic position. This further underlines their likely competition. Apart from that, Marentette et al. (2010) suggested that round goby can be a vector of contaminants to the food web. Our study does not indicate that both invasive gobies and native ruffes tend to transfer metals to higher trophic levels.

We conclude that the combination of a traditional food web study based on analyses of nitrogen and carbon isotopes with the analyses of additional elements, such as metals, could provide (additional) insights in biomagnification or biodilution and trophic linkages and/or niche similarities within a system. Overall, our study illustrates that (heavy) metal contents of species can differ between feeding guilds and/or species, and correlate with food web trophy (measured as δ^15^N). Organisms with higher metal contents are found lower in the food web. The differences can be attributed to metabolic and physiological processes. The heavy metals did not biomagnify in the food web and within the fish species biodilution occurs. Although it was not possible to elucidate trophic linkages between species based on solely heavy metal or other elemental contents, the elemental signatures did reveal some niche or habitat similarities and differences between organisms/taxonomic groups. This approach of combining isotopic signatures with elemental analyses might be helpful in other systems as well or in hindsight, metal analyses can be used to obtain information on food web structure where no isotopic signatures are available.

### Supplementary Information

Below is the link to the electronic supplementary material.

Supplementary file1 (DOCX 1551 kb)

## Data Availability

The dataset generated during and/or analysed during the current study will become available in the DANS-EASY repository: https://doi.org/10.17026/dans-zzt-9xs3.
