# Supplementary material for: Trophic Transfer of Cd, Cu, Pb, Zn, P and Se in Dutch Storage Water Reservoirs
Source: Arch Environ Contam Toxicol. 2024 Jan 20;86(3):217–33. doi: 10.1007/s00244-023-01041-x (PMC11032288; doi:10.1007/s00244-023-01041-x)
Supplement: Supplementary file 1 — Supplementary file1 (DOCX 1551 kb) [file 244_2023_1041_MOESM1_ESM.docx]

**Supplementary Information for**

**Trophic transfer of Cd, Cu, Pb, Zn, P and Se in Dutch storage water reservoirs**

In: *Archives of Environmental Contamination and Toxicology*

Verstijnen, Y.J.M.^*^, Lucassen, E.C.H.E.T., Wagenvoort, A.J., Ketelaars, H. A. M., van der Velde, G. and A.J.P. Smolders

*Corresponding author. B-WARE Research Centre, Radboud University, Nijmegen, The Netherlands. Department of Aquatic Ecology and Environmental Biology, Radboud Institute for Biological and Environmental Sciences (RIBES), Radboud University, Nijmegen, The Netherlands. (e-mail: y.verstijnen@b-ware.eu)

**Tables**

**Table SI1** Average concentrations per year of Total-P, Cd, Cu, Pb, Se and Zn in the water of the River Meuse (at Keizersveer 10 km upstream of the reservoirs) and in the water after reservoir retention (outflow Petrusplaat)

|  |  | µg/l | µg/l | µg/l | µg/l | µg/l | µg/l |
| --- | --- | --- | --- | --- | --- | --- | --- |
| Year | Location | Tot-P | Cd | Cu | Pb | Se | Zn |
| 2010 | River Meuse | 152.78 | 0.11 | 6.17 | 2.31 | 0.54 | 15.92 |
| 2010 | Outflow Petrusplaat | 53.26 | 0.02 | 2.17 | 0.62 | 0.29 | 2.20 |
| 2011 | River Meuse | 134.15 | 0.13 | 2.69 | 1.56 | 0.41 | 13.39 |
| 2011 | Outflow Petrusplaat | 55.53 | 0.06 | 2.32 | 0.40 | 0.31 | 3.57 |
| 2012 | River Meuse | 142.47 | 0.13 | 2.49 | 2.29 | 0.23 | 13.88 |
| 2012 | Outflow Petrusplaat | 61.16 | 0.06 | 2.39 | 0.27 | 0.21 | 3.09 |
| 2013 | River Meuse | 126.42 | 0.09 | 2.16 | 1.33 | 0.23 | 10.99 |
| 2013 | Outflow Petrusplaat | 62.21 | 0.05 | 1.83 | 0.11 | 0.21 | 2.47 |
| 2014 | River Meuse | 142.84 | 0.16 | 2.70 | 2.63 | 0.24 | 16.57 |
| 2014 | Outflow Petrusplaat | 58.04 | 0.05 | 3.03 | 0.21 | 0.22 | 2.75 |
| 2015 | River Meuse | 127.11 | 0.12 | 2.61 | 1.99 | 0.26 | 14.38 |
| 2015 | Outflow Petrusplaat | 47.08 | 0.04 | 2.02 | 0.17 | 0.22 | 2.40 |
| 2016 | River Meuse |  | 0.09 | 2.13 | 1.25 | 0.25 | 10.55 |
| 2016 | Outflow Petrusplaat |  | 0.04 | 1.95 | 0.18 | 0.23 | 2.34 |
| 2017 | River Meuse |  | 0.09 | 2.38 | 1.08 | 0.26 | 11.19 |
| 2017 | Outflow Petrusplaat |  | 0.04 | 1.78 | 0.17 | 0.21 | 3.26 |
| 2018 | River Meuse | 110.85 | 0.14 | 2.81 | 1.53 | 0.36 | 11.78 |
| 2018 | Outflow Petrusplaat | 43.19 | 0.03 | 3.53 | 0.31 | 0.37 | 3.66 |
| 2019 | River Meuse | 110.18 | 0.08 | 2.83 | 1.43 | 0.29 | 8.67 |
| 2019 | Outflow Petrusplaat | 29.07 | 0.03 | 1.80 | 0.09 | 0.24 | 3.54 |

**Table SI2** sample size (n) and elemental content (µg/g DW) of the sediment in Petrusplaat

|  | **n** | **Al** | **As** | **Ca** | **Cd** | **Co** | **Cr** | **Cu** | **Fe** | **K** | **Mg** |
| --- | --- | --- | --- | --- | --- | --- | --- | --- | --- | --- | --- |
| Sediment Petrusplaat | 1 | 768 | 5.2 | 349808 | 0.9 | 1.1 | 4.0 | 5.2 | 1046 | 148 | 1441 |
|  |  |  | **Mn** | **Na** | **Ni** | **P** | **Pb** | **S** | **Si** | **Sr** | **Zn** |
|  |  |  | 196 | 231 | 3.7 | 694 | 6.0 | 1685 | 566 | 423 | 74 |

**Table SI3** Sampling methods and if applicable size and fresh weight ranges, per species or organism-group (adjusted from Verstijnen et al. (2019))

|  | **Sample method** | **Size range (mm)** | **Fresh weight (g)** | **Depth (m)** |
| --- | --- | --- | --- | --- |
| **Algae/Cyanobacteria** |  |  |  |  |
| Benthic cyanobacteria | Manual (from sediment collected with Eckman grabber or by scuba diver) |  |  | <6 |
| *Chara contraria* | Manual (from the shore and snorkeling) |  |  | 0 and 5 |
| Cyanobacteria (diverse species) | Tow net (30 and 100 µm), lifted out of surface water |  |  |  |
|  |  |  |  |  |
| Seston | Tow net (30, 100 and 250 µm) |  |  |  |
| Thread-algae (*Vaucheria* sp., *Enteromorpha* sp.) | Manual (snorkeling or from the shore) |  |  | 3 |
|  |  |  |  |  |
| **Macrophytes** |  |  |  |  |
| Curled pondweed (*Potamogeton crispus*) | Manual (snorkeling, by scuba diver or from the shore) |  |  | <6 |
| Perfoliate pondweed (*Potamogeton perfoliatus*) | Manual (snorkeling) |  |  | 6 |
| Sago pondweed (*Stuckenia pectinata* formerly *P. pectinatus*) | Manual (snorkeling, by scuba diver or from the shore) |  |  | <6 |
| Water weed (*Elodea nuttallii*) | Collected with Eckman grabber or Manual (by scuba diver, snorkeling or out of net) |  |  | 3-6 |
| Watermilfoil (*Myriophyllum spicatum*) | Manual (snorkeling or by scuba diver). |  |  | 3-6 |
|  |  |  |  |  |
| **Zooplankton** |  |  |  |  |
|  |  |  |  |  |
| Zooplankton (i.a *Eudiaptomus*, *nauplius* larvae, *Cyclops*) | With plankton net (30, 100 and/or 250 µm). Or manually found in other samples and separated. |  |  |  |
|  |  |  |  |  |
| **Fish** |  |  |  |  |
| Bream (*Abramis brama*) | Net (multimesh gillnet or seine) | 415-605 | 1630-4600 | |
| Fish larvae | Trawl |  |  |  |
| Perch (*Perca fluviatilis*) | Net (multimesh gillnet or seine) | 45-415 | 1.4-1892.6 | |
| Pike-perch (*Sander lucioperca*) | Net (multimesh gillnet or seine) | 56-760 | 2-6140 |  |
| Roach (*Rutilus rutilus*) | Net (multimesh gillnet or seine) | 53-118 | 3.1-35.0 |  |
| Round goby (*Neogobius melanostomus*) | Net (multimesh gillnet or seine) | 48-150 | 1.8-90.1 |  |
| Ruffe (*Gymnocephalus cernuus*) | Net (multimesh gillnet or seine) | 47-152 | 2.3-34.7 |  |
| Smelt (*Osmerus eperlanus*) | Net (multimesh gillnet or seine) | 75 | 3.9 |  |
| Whitefish (*Coregonus* sp.) | Net (multimesh gillnet or seine) | 125-460 | 25.6-1864 |  |
|  |  |  |  |  |
| **Macroinvertebrates and other** |  |  |  |  |
|  |  |  |  |  |
| Crayfish (*Faxonius limosus*) | Seine fishing |  |  |  |
| Fresh water jellyfish (*Craspedacusta sowerbyi*) | Manual (surface water) |  |  |  |
| Gammarid (*Dikerogammarus* sp.) | Manual (scuba diver, snorkelling or found in other samples), net |  |  |  |
| Detritus mussel banks | Excreted by quagga mussels during 4 hours |  |  |  |
| Sediment | Van Veen sampler |  |  |  |
| Sponges | Manual (scuba diver) |  |  |  |
| Quagga-mussel (*Dreissena rostriformis bugensis*) | Sediment sampler, trawl or manual (snorkelling) | 16.33-31.61 | 0.13-1.21 | 3-6 |

**Table SI4** Typical instrumental limits of detection (LOD in ppb) of the ICP-OES and ICP-MS equipment.

|  | ICP-OES | ICP-MS |
| --- | --- | --- |
|  | ppb | ppb |
| Al | 1.51 |  |
| As | 4.74 | 0.121 |
| Ca | 0.02 |  |
| Cd | 0.19 | 0.0002 |
| Co | 1.16 | 0.0002 |
| Cr | 0.85 | 0.012 |
| Cu | 2.36 | 0.0008 |
| Fe | 0.80 |  |
| K | 5.10 |  |
| Mg | 0.04 |  |
| Mn | 0.21 |  |
| Na | 1.80 |  |
| Ni | 2.29 | 0.002 |
| P | 5.66 |  |
| Pb | 4.50 | 0.00007 |
| S | 2.22 |  |
| Se | 7.36 | 0.031 |
| Si | 7.20 |  |
| Sr | 0.04 |  |
| Zn | 0.60 | 0.00290 |

**Table SI5** sample size (n) and median elemental content (µg/g DW) per species or taxon

|  | **n** | **Al** | **As*** | **Ca** | **Cd*** | **Co** | **Cr** | **Cu** | **Fe** | **K** | **Mg** | **Mn** | **Na** | **Ni** | **P** | **Pb** | **S** | **Se** | **Si** | **Sr** | **Zn** |
| --- | --- | --- | --- | --- | --- | --- | --- | --- | --- | --- | --- | --- | --- | --- | --- | --- | --- | --- | --- | --- | --- |
| *Abramis brama* >40 cm | 7 | 551 | 0.7 | 448 | 0.1 | 0.1 | 0.8 | 0.9 | 27 | 13659 | 1104 | 0.7 | 1141 | 0.3 | 8028 | 0.3 | 8318 | 1.4 | 58 | 2.6 | 12 |
| Benthic cyanobacteria | 3 | 919 | 1.4 | 43390 | 0.5 | 0.9 | 9.0 | 7.9 | 1729 | 2806 | 2317 | 211.8 | 954 | 14.3 | 2001 | 15.1 | 1870 | 0.7 | 390 | 259.1 | 70 |
| *Chara contraria* | 7 | 440 | 1.3 | 111490 | 0.4 | 0.5 | 0.8 | 3.4 | 152 | 5962 | 3054 | 69.9 | 1325 | 10.8 | 1506 | 1.2 | 2668 | 0.9 | 284 | 232.7 | 26 |
| *Coregonus* sp. <15 cm | 9 | 212 | 0.6 | 1556 | 0.1 | 0.1 | 0.5 | 1.0 | 30 | 16180 | 1367 | 1.3 | 1036 | 0.3 | 11096 | 0.1 | 8704 | 0.9 | 33 | 4.2 | 27 |
| *Coregonus* sp. >29 cm | 9 | 762 | 0.5 | 347 | 0.1 | 0.1 | 0.6 | 0.6 | 22 | 13455 | 1159 | 0.8 | 757 | 0.2 | 9246 | 0.1 | 8107 | 0.9 | 18 | 1.9 | 14 |
| *Craspedacusta sowerbyi* | 1 | 295 | 2.9 | 7023 | 2.5 | 0.4 | 1.8 | 8.4 | 213 | 3927 | 2159 | 8.6 | 17807 | 3.2 | 11466 | 2.7 | 8239 | 1.9 | 194 | 23.8 | 118 |
| Cyanobacteria | 9 | 337 | 4.4 | 16752 | 1.2 | 1.1 | 1.4 | 12.7 | 348 | 6712 | 3433 | 35.4 | 1270 | 14.6 | 6854 | 2.2 | 6722 | 0.4 | 860 | 67.9 | 38 |
| Detritus mussel banks | 2 | 13452 | 9.5 | 79956 | 5.4 | 24.5 | 56.7 | 283.8 | 27218 | 2290 | 5485 | 3289.3 | 1829 | 53.1 | 1966 | 134.0 | 1943 | 1.9 | 4849 | 162.3 | 644 |
| *Dikerogammarus* sp. | 11 | 1345 | 1.5 | 120333 | 0.2 | 0.3 | 1.4 | 27.9 | 164 | 2964 | 3933 | 46.4 | 3796 | 5.3 | 5597 | 1.2 | 3012 | 3.0 | 597 | 241.4 | 88 |
| *Dreissena rostriformis bugensis* | 41 | 508 | 6.1 | 4039 | 3.8 | 1.2 | 1.8 | 14.5 | 538 | 1425 | 822 | 81.6 | 1503 | 5.4 | 7724 | 3.0 | 6470 | 2.8 | 498 | 17.0 | 88 |
| *Elodea nuttallii* | 14 | 934 | 1.1 | 25622 | 1.3 | 1.9 | 1.2 | 17.0 | 554 | 23257 | 1572 | 312.1 | 3791 | 16.7 | 3480 | 3.6 | 2484 | 1.3 | 882 | 71.9 | 103 |
| *Enteromorpha* sp. | 8 | 872 | 1.2 | 62454 | 0.2 | 0.2 | 0.8 | 3.8 | 138 | 6900 | 4218 | 33.0 | 1971 | 5.2 | 614 | 2.2 | 22379 | 1.1 | 547 | 159.2 | 22 |
| *Faxonius limosus* | 2 | 465 | 2.0 | 5454 | 0.1 | 0.2 | 0.8 | 131.7 | 43 | 10189 | 1156 | 3.0 | 9615 | 2.2 | 8180 | 0.3 | 8660 | 1.1 | 55 | 15.0 | 315 |
| Fish larvae | 2 | 2031 | 2.8 | 59708 | 0.5 | 2.5 | 3.4 | 28.3 | 392 | 8994 | 2535 | 61.8 | 2799 | 7.7 | 14869 | 2.4 | 7041 | 1.3 | 566 | 86.9 | 187 |
| *Gymnocephalus cernuus* <6 cm | 11 | 829 | 0.9 | 5002 | 0.1 | 0.2 | 0.8 | 1.1 | 42 | 14971 | 1457 | 2.1 | 2002 | 0.6 | 11404 | 0.2 | 10136 | 1.7 | 72 | 7.2 | 35 |
| *Gymnocephalus cernuus* >8 cm | 16 | 475 | 1.0 | 1093 | 0.1 | 0.2 | 0.7 | 0.9 | 32 | 14876 | 1465 | 2.2 | 964 | 0.6 | 9231 | 0.3 | 10214 | 2.0 | 58 | 4.0 | 25 |
| *Myriophyllum spicatum* | 6 | 539 | 0.8 | 18189 | 0.6 | 0.8 | 1.1 | 8.1 | 367 | 10501 | 1612 | 181.6 | 9094 | 4.8 | 2613 | 3.0 | 2718 | 2.0 | 533 | 54.7 | 81 |
| *Neogobius melanostomus* | 43 | 264 | 1.0 | 3582 | 0.1 | 0.1 | 0.8 | 1.3 | 28 | 15267 | 1439 | 2.5 | 1613 | 0.5 | 10225 | 0.3 | 10052 | 1.4 | 56 | 3.5 | 23 |
| *Osmerus eperlanus* | 1 | 606 | 1.3 | 1974 | 0.1 | 0.1 | 0.7 | 1.8 | 23 | 12321 | 1189 | 2.4 | 3506 | 0.2 | 9168 | 0.4 | 9280 | 0.9 | 23 | 2.7 | 33 |
| *Perca fluviatilis* <15 cm | 15 | 340 | 0.9 | 4962 | 0.1 | 0.2 | 0.9 | 1.3 | 50 | 16363 | 1427 | 2.5 | 1459 | 0.7 | 12236 | 0.2 | 10387 | 1.0 | 66 | 7.0 | 35 |
| *Perca fluviatilis* >30 cm | 11 | 246 | 0.7 | 518 | 0.1 | 0.1 | 0.7 | 0.7 | 23 | 16132 | 1309 | 1.0 | 1344 | 0.5 | 9944 | 0.1 | 10476 | 1.6 | 32 | 2.5 | 18 |
| *Perca fluviatilis* 15-30 cm | 21 | 215 | 0.7 | 579 | 0.1 | 0.1 | 0.6 | 0.8 | 30 | 16949 | 1387 | 1.1 | 1345 | 0.4 | 10354 | 0.1 | 11114 | 1.2 | 50 | 2.7 | 22 |
| *Potamogeton crispus* | 11 | 367 | 0.5 | 16278 | 0.5 | 0.8 | 0.7 | 11.5 | 203 | 15984 | 1368 | 55.8 | 8474 | 5.5 | 3297 | 1.5 | 2894 | 1.6 | 462 | 54.7 | 51 |
| *Stuckenia pectinata*  *(Potamogeton pectinatus)* | 7 | 405 | 0.7 | 14719 | 0.2 | 0.3 | 0.9 | 8.7 | 130 | 18204 | 3205 | 29.6 | 8530 | 5.6 | 3264 | 3.3 | 4664 | 0.8 | 663 | 49.7 | 48 |
| *Potamogeton perfoliatus* | 4 | 392 | 0.5 | 16377 | 0.5 | 0.7 | 1.1 | 11.3 | 127 | 13028 | 3666 | 61.9 | 5092 | 8.1 | 2453 | 2.2 | 4970 | 2.7 | 472 | 59.2 | 69 |
| *Rutilus rutilus* <12 cm | 12 | 401 | 1.4 | 3216 | 0.1 | 0.3 | 0.9 | 1.5 | 51 | 15740 | 1485 | 1.9 | 879 | 0.9 | 11778 | 0.2 | 8983 | 0.6 | 157 | 10.1 | 50 |
| *Sander lucioperca* <20 cm | 34 | 288 | 1.0 | 1801 | 0.1 | 0.1 | 0.7 | 1.0 | 29 | 16278 | 1400 | 1.5 | 1826 | 0.4 | 9870 | 0.2 | 11704 | 0.9 | 37 | 3.4 | 25 |
| *Sander lucioperca* >40 cm | 7 | 440 | 0.6 | 688 | 0.1 | 0.1 | 0.8 | 0.7 | 21 | 15659 | 1230 | 0.7 | 1419 | 0.3 | 8964 | 0.1 | 12866 | 1.1 | 29 | 0.8 | 19 |
| *Sander lucioperca* 20-30 cm | 3 | 195 | 0.8 | 577 | 0.1 | 0.0 | 0.7 | 0.6 | 18 | 16720 | 1386 | 0.7 | 1259 | 0.3 | 9474 | 0.2 | 12943 | 1.2 | 29 | 2.7 | 17 |
| Seston | 7 | 1934 | 3.2 | 24409 | 1.3 | 3.3 | 10.0 | 160.7 | 1970 | 7133 | 2739 | 147.2 | 5073 | 55.7 | 6373 | 25.1 | 7426 | 0.7 | 5599 | 77.0 | 662 |
| Sponges | 1 | 280 | 1.1 | 16050 | 6.7 | 0.3 | 1.2 | 5.5 | 816 | 901 | 772 | 25.5 | 795 | 3.4 | 5267 | 2.6 | 2997 | 2.1 | 939 | 26.4 | 62 |
| *Vaucheria* sp. | 5 | 1174 | 3.8 | 49645 | 0.7 | 3.2 | 3.9 | 10.1 | 1503 | 9244 | 1767 | 557.6 | 1129 | 9.4 | 2465 | 7.9 | 10577 | 1.3 |  | 73.0 | 91 |
| Zooplankton | 13 | 6411 | 5.4 | 29806 | 1.7 | 7.4 | 18.7 | 80.8 | 6424 | 4758 | 2962 | 397.4 | 1657 | 40.4 | 6740 | 42.9 | 6003 | 1.9 | 2814 | 127.7 | 329 |

*medians were calculated based on the adjusted values, see details in the methods.

**Figures**


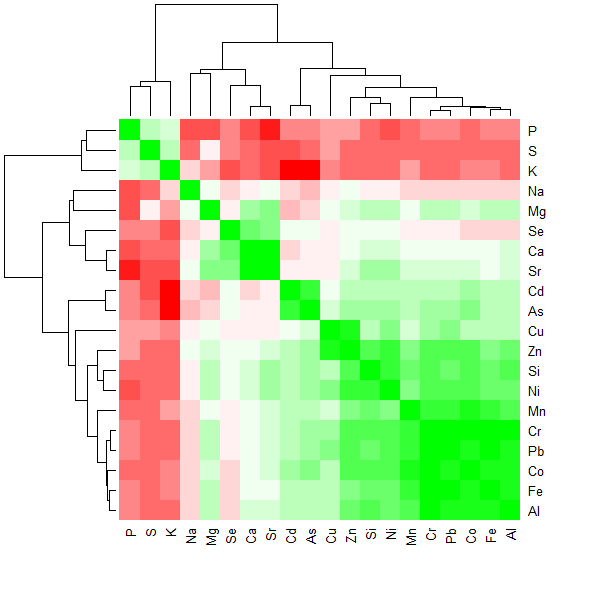


**Fig. SI1** Heatmap of correlations between elements, based on all samples. Red = negative correlation, green = positive correlation. The brighter or more intense the colour, the stronger the correlation. Bright red: r = -1. Bright green: r = 1


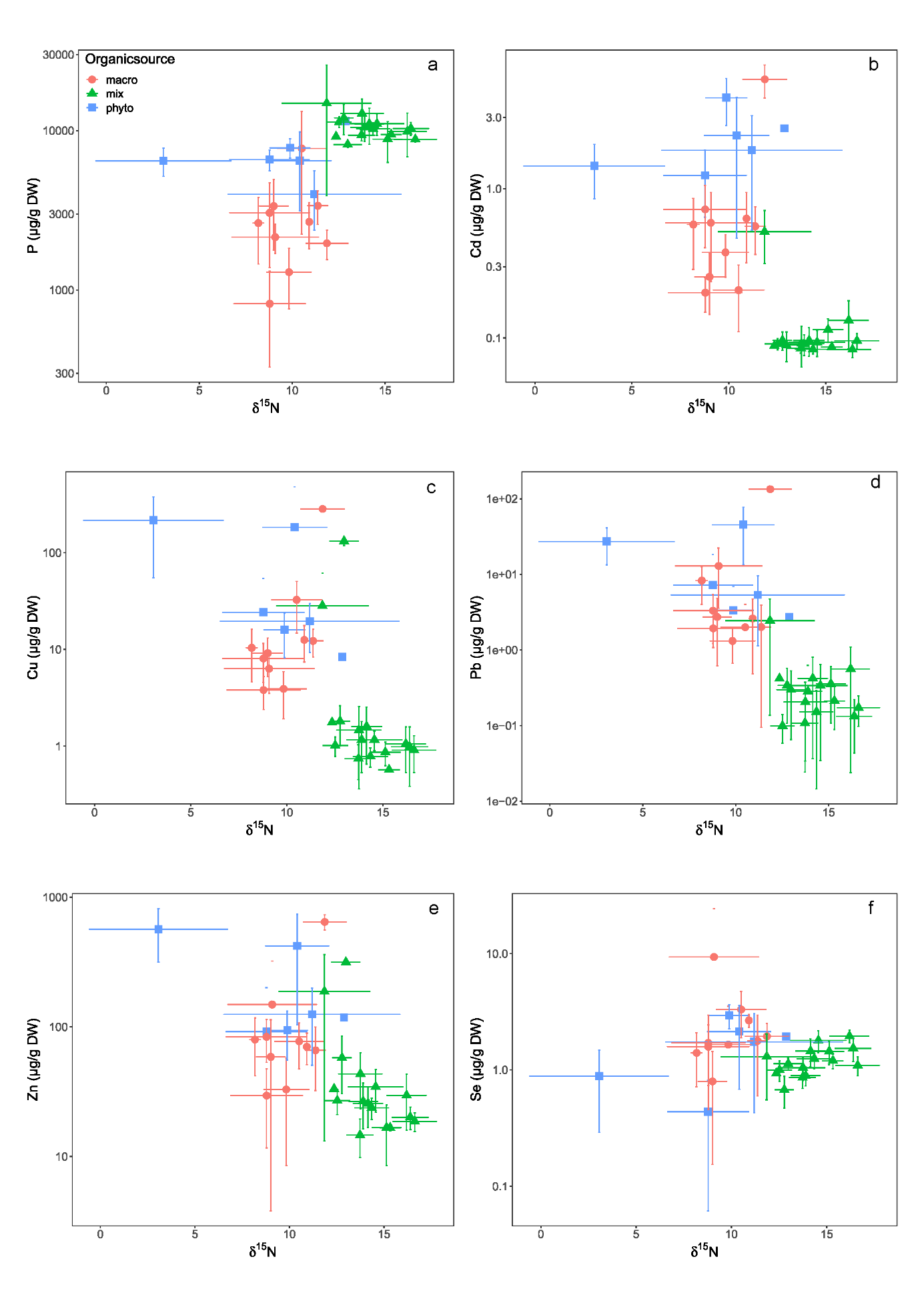


**Fig. SI2** Average elemental contents (µg/g dry weight ± standard deviation; on a log-scale) of the selected relevant elements (depicted from the different clusters in Fig. 2) versus δ^15^N, grouped per organism/taxon and coloured by organic source of the organism (macrophytes, phytoplankon or mix, based on δ^13^C; stable isotope ratios from Verstijnen et al. 2019). A: phosphorus, B: cadmium, C: copper, D: lead, E: selenium and F: zinc





**Fig. SI3** Cadmium (A) and zinc (B) contents (µg/g dry weight) per fish species related to fish length (mm)
